# Early Menarche as a Risk Factor for Suicidality in Female Patients

**DOI:** 10.1192/j.eurpsy.2026.11979

**Published:** 2026-07-31

**Authors:** K. Laškarin, J. Kosor Ljoka, E. Ivezić, K. Matić, N. Ruljančić, V. Grošić, I. Filipčić, S. Vuk Pisk

**Affiliations:** 1 University Psychiatric Hospital Sveti Ivan, Zagreb; 2 Faculty of Dental Medicine and Health Osijek, Osijek; 3 The University of Zagreb School of Medicine; 4 University of Applied Health Sciences, Zagreb, Croatia

## Abstract

**Introduction:**

Early adolescence represents the developmental period with the highest incidence of suicidal ideation, particularly among girls, coinciding with the onset of menarche as the final stage of puberty. Evidence indicates that younger age at menarche increases vulnerability to psychopathology and has been specifically linked to higher rates of suicidal thoughts and behaviors. The developmental readiness hypothesis suggests that early maturers face biological and social demands prematurely, which may overwhelm coping capacities and contribute to maladjustment. Hormonal influences, particularly interactions of ovarian hormones with the hypothalamic–pituitary–adrenal (HPA) axis and serotonergic systems, may additionally heighten emotional reactivity and suicidal risk.

**Objectives:**

To investigate the relationship between age at menarche and the intensity of suicidality in female patients with psychiatric disorders.

**Methods:**

A total of 85 female patients aged 18–71 years (mean = 41.02, SD = 15.66) were included in a prospective study. Primary psychiatric diagnoses included depressive disorder (62.4%), anxiety-depressive disorder (14.1%), bipolar disorder (9.4%), schizoaffective disorder (4.7%), adjustment disorder (3.5%), and borderline personality disorder (1.2%). The mean age at menarche was 13.02 years (SD = 1.73, range 9–18). Participants completed a demographic questionnaire and standardized scales, with suicidality assessed using the Suicide Behaviors Questionnaire-Revised (SBQ-R).

**Results:**

Earlier age at menarche was significantly associated with higher total scores on the SBQ-R, indicating greater overall severity of suicidality compared to those with normative or later menarcheal timing.

**Conclusions:**

The findings highlight early menarche as a potential risk factor for increased suicidality in women with psychiatric disorders. These results emphasize the importance of early identification of girls with early menarche as a vulnerable group, and support the implementation of timely preventive and therapeutic interventions targeting suicide risk and emotional regulation.

**Disclosure of Interest:**

None Declared

